# Anti-Inflammatory Effect of Topiramate in a Chronic Model of TNBS-Induced Colitis

**DOI:** 10.3390/ijms23169127

**Published:** 2022-08-15

**Authors:** Inês Silva, Priscila Mendes, Sofia Guerra, Rui Pinto, Vanessa Mateus

**Affiliations:** 1 H&TRC–Health and Technology Research Center, ESTeSL–Lisbon School of Health and Technology, Instituto Politécnico de Lisboa, 1990-096 Lisbon, Portugal; 2 iMed.ULisboa, Faculdade de Farmácia, Universidade de Lisboa, 1990-096 Lisbon, Portugal; 3 JCS, Dr. Joaquim Chaves, Laboratório de Análises Clínicas, Miraflores, 1495-069 Algés, Portugal

**Keywords:** inflammatory bowel disease, TNBS-induced colitis, topiramate, anti-inflammatory effect, experimental colitis model

## Abstract

Inflammatory bowel disease (IBD) is characterized by a chronic and relapsing inflammatory response in the gastrointestinal tract, resulting in severe symptoms such as abdominal pain, vomiting, diarrhea, bloody stools, and weight loss. Currently, there is no cure, and the pharmacological treatment includes drugs that induce and keep the patient in remission, not reversing the underlying pathogenic mechanism. These therapies, in the long term, may cause various side effects and complications, which has increased the need to investigate new, more effective, and safer pharmacological approaches. In preclinical studies, topiramate has demonstrated a potential anti-inflammatory effect by inhibiting the production of several pro-inflammatory cytokines. This study aimed to investigate the effect of topiramate in a chronic TNBS-induced colitis model in rodents. Experimental colitis was induced by four intrarectal administrations of 1% TNBS in female CD-1 mice. Topiramate 10 and 20 mg were administered intraperitoneally for 14 days. Several parameters were evaluated, such as bodyweight, alkaline phosphatase (ALP), fecal hemoglobin, fecal calprotectin, tumor necrosis factor (TNF)-α, and interleukin (IL)-10. Topiramate reduces TNBS-induced colonic damage in a model of chronic experimental colitis and normalizes the stool consistency and anus appearance. Additionally, topiramate significantly reduced the concentration of ALP, fecal hemoglobin, fecal calprotectin, TNF-α, and IL-10, demonstrating it to be a promising pharmacological approach for the treatment of IBD in the future.

## 1. Introduction

Inflammatory bowel disease (IBD) is a chronic inflammatory disorder of the gastrointestinal (GI) tract that comprises two primary forms: Crohn’s disease (CD) and ulcerative colitis (UC) [1,2,3,4]. CD is manifested by a transmural and discontinuous ulceration of any portion of the GI tract, mainly affecting the terminal ileum and the perianal region [2,4]. UC is manifested by continuous ulceration of the mucosa and may affect part of the colon or the entire colon [1,4]. Currently, IBD affects more than 0.5% of the population in westernized countries with the highest prevalence in Europe, where UC is the most prevalent—it affects approximately 505 out of 100,000 people [5,6]. Nowadays, there is no cure for this disease, which is quite debilitating for the patient. Current pharmacological treatment options for IBD are based on controlling the symptoms, reducing the periods of active disease, and maintaining remission [7].

Topiramate is an antiepileptic and anticonvulsant drug used as a first-line drug in the treatment of seizure disorders, mainly primary generalized-onset tonic-clonic seizures and partial-onset seizures. This drug was initially approved by the FDA in 1996 for the treatment of monotherapy epilepsy, adjunctive therapy epilepsy, and migraine disorder, showing some antidepressant activity [8,9]. According to the literature, topiramate also has anti-inflammatory properties. Its exact mechanism of action remains unclear, but it is known to potentiate chloride channels activated by gamma-aminobutyric acid (GABA). GABA receptors, located in the GI tract, are responsible for motility, secretion, and immunity, which makes topiramate a potentially useful target in IBD [10]. In addition, this drug showed activity on AMPA/kainate receptors. Preclinical studies have shown that topiramate inhibits the production of several proinflammatory cytokines, among them interleukin(IL)-17, IFN-γ, tumor necrosis factor(TNF)-α, IL-6, and IL-10, and have demonstrated that this drug can act on T cells or antigen-presenting cells (APC), suppressing inflammatory signaling [9,10,11]. Since topiramate already has a marketing authorization (AIM) and is considered a safe and effective drug for the treatment of neurological diseases in humans, it would be very useful to assign it as repurposing for its use in IBD. In addition, topiramate has a more beneficial profile when compared to the drugs currently used in the treatment of IBD, which raises the possibility that this drug may prove to be a promising new pharmacological alternative [12]. Thus, the overall aim of this study is to evaluate the effect of topiramate in IBD through an animal model of TNBS-induced chronic colitis, in order to provide more effective treatment with fewer adverse effects.

## 2. Results

### 2.1. Clinical Signs

During 6 weeks, the mice were monitored daily for body weight, stool consistency, anus appearance, and survival rate. Throughout the experimental process, the animals with TNBS-induced colitis presented changes in intestinal motility, characterized by the presence of diarrhea and/or soft stool, allied with moderate edema of the anus. In the experimental groups treated with topiramate 10mg (TNBS + TOP10) and topiramate 20 mg (TNBS + TOP20), topiramate has been shown to ameliorate stool consistency and anus appearance.

**Figure 1:**
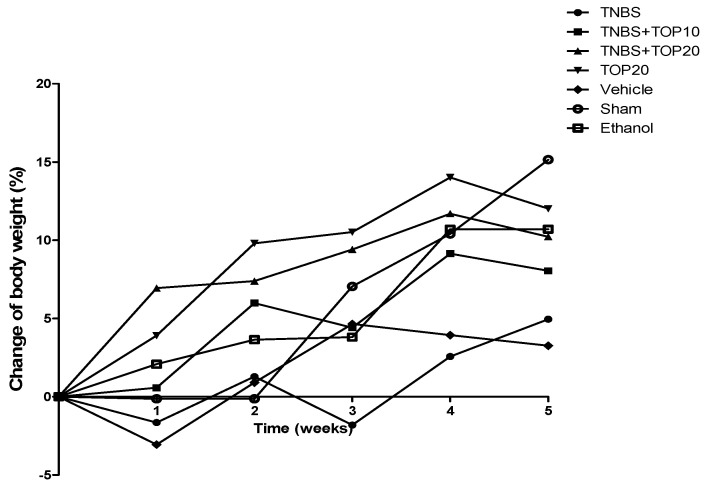
Change in bodyweight during the experimental study.

The body weight was measured throughout the experimental process (Figure 1), and an increase in all experimental groups was noticed, although with some variances, until the end of the experiment. The animals of the sham group presented a higher increase in their body weight, gaining 15.15 ± 1.06% in six weeks. Contrariwise, the TNBS and the vehicle groups showed a lower increase with a mean variation of −0.89 ± 1.06% and −1.62 ± 1.19%, respectively. Between TNBS + TOP10 and TNBS + TOP20 groups, they presented a variation of 8.05 ± 1.55% and 10,23 ± 1.69% at the end of the experiment. In the TOP20 and ethanol control groups, it was observed a variation in their bodyweights of 8.38 ± 2.18%, and 5.16 ± 1.84%, respectively. There were no significant differences between all groups. Therefore, it is not possible to determine a significant effect of TOP in the variation of the bodyweight between the treated and non-treated groups.

### 2.2. Biochemical Markers

**Figure 2:**
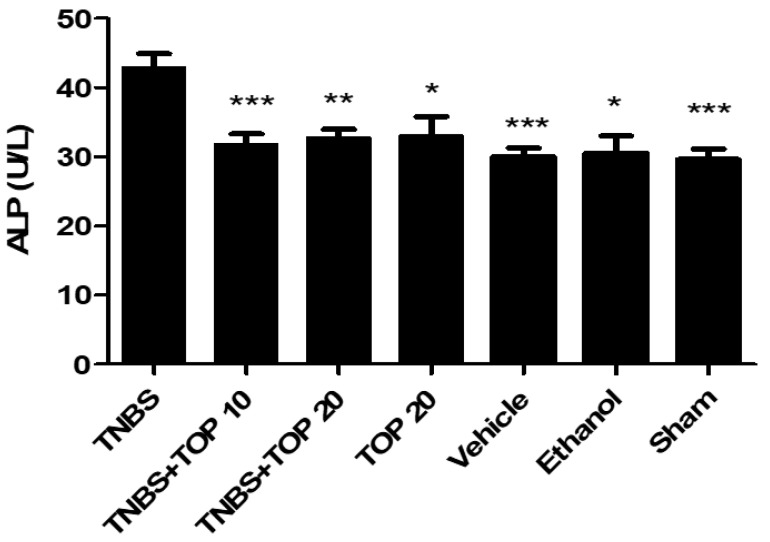
Effect of topiramate treatment on ALP concentration. Legend: one-way ANOVA and Tukey’s post-hoc test, * *p* < 0.01 compared with the TNBS group ** *p* < 0.001 compared with the TNBS group, *** *p* < 0.0001 compared with the TNBS group.

Due to its essential role in intestinal homeostasis, the concentration of alkaline phosphatase (ALP) in the blood was evaluated (Figure 2) [13]. As expected, the highest concentration of ALP was noticed in the TNBS group (42.88 ± 2.01 U/L). The ALP values presented statistically significant differences in the TNBS group compared to the treated groups, with the TNBS + TOP10 group (31.75 ± 1.54 U/L, *p <* 0.0001) and the TNBS + TOP20 group (32.75 ± 1.24 U/L), *p <* 0.001) showing a beneficial effect of topiramate in terms of the expression of intestinal ALP. Furthermore, the TNBS + TOP10 and TNBS + TOP20 groups produced results that were very similar to the control groups TOP20 (33.00 ± 2.80 U/L), vehicle (30.00 ± 1.29 U/L), sham (29.60 ± 1.54 U/L), and ethanol (30.50 ± 2.50 U/L).

**Figure 3:**
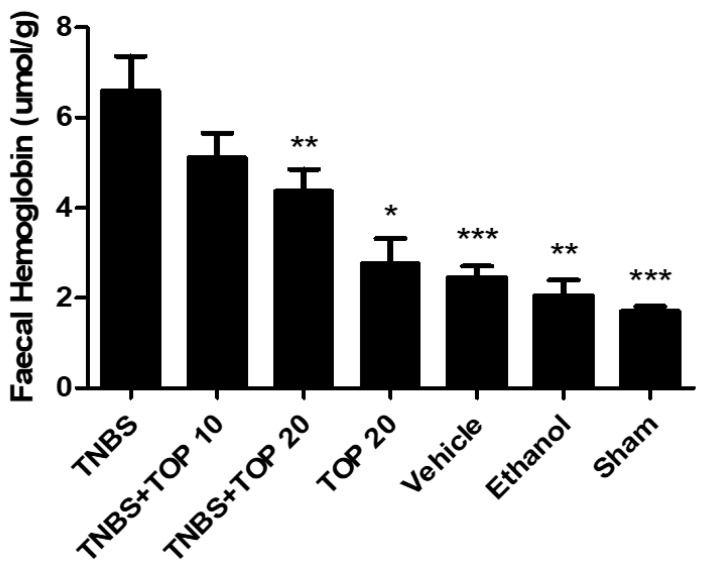
Effect of topiramate treatment on fecal hemoglobin concentration. Legend: one-way ANOVA and Tukey’s post-hoc test, * *p* < 0.01 compared with the TNBS group.** *p* < 0.001 compared with the TNBS group, *** *p* < 0.0001 compared with the TNBS group.

Fecal hemoglobin is an important marker that allows for the evaluation of the intensity of hemorrhagic focus and the comparison and analysis of the severity of colitis lesions between the experimental groups [14]. Thus, fecal hemoglobin values were recorded in all experimental groups (Figure 3). The TNBS group (6.60 ± 0.76 μmol/g) had a significantly higher concentration of fecal hemoglobin in comparison with the control groups TOP20 (2.78 ± 0.55 μmol/g, *p <* 0.01), ethanol (2.05 ± 0.35 μmol/g, *p <* 0.001), vehicle (2.45 ± 0.26 μmol/g, *p* < 0.0001), and sham (1.70 ± 0.11 μmol/g, *p* < 0.0001. Topiramate demonstrated a positive influence on this parameter but only in the highest dose (20 mg) with significant differences between the TNBS and the TNBS + TOP20 groups (6.60 ± 0.76 μmol/g vs. 4.38 ± 0.48 μmol/g, *p* < 0.001). However, it is not possible to determine a dose-dependent effect since there are no statistically significant differences between both groups treated with topiramate.

**Figure 4:**
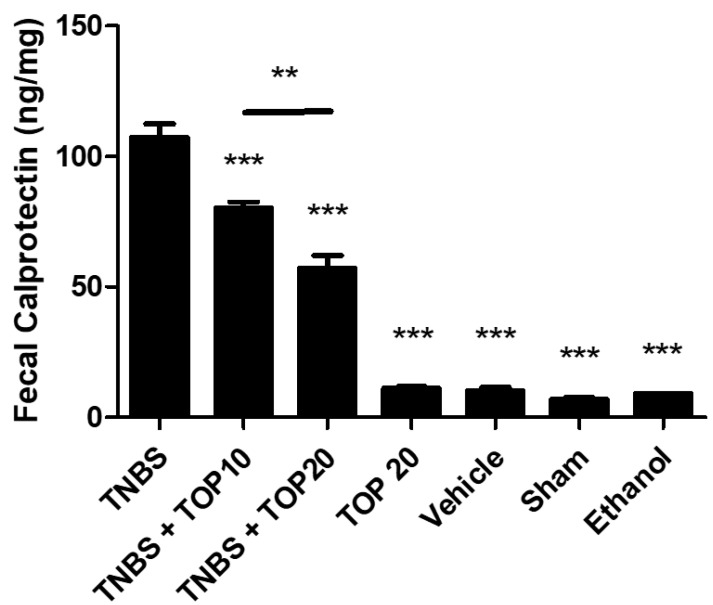
Effect of topiramate treatment on fecal calprotectin concentration. Legend: one-way ANOVA and Tukey’s post-hoc test, ** *p* < 0.001 between groups, *** *p* < 0.0001 compared with the TNBS group.

Fecal calprotectin is an important marker related to mucosal inflammation, and its concentration is directly associated with the leukocyte accumulation in stool [15]. In this sense, in order to evaluate the influence of TOP in this marker, it was quantified and compared between all the groups (Figure 4). As expected, the TNBS group revealed the highest concentration of fecal calprotectin, namely 107.30 ± 5.24 ng/mg. The values obtained in TNBS + TOP10 (80.13 ± 2.54 ng/mg) and TNBS + TOP20 (57.00 ± 5.12 ng/mg) groups revealed that the treatment with TOP had the capability to significantly reduce the concentration of this marker, in comparison to the TNBS group (*p* < 0.001). It is also possible to determine a dose-dependent effect by TOP since there are significant differences (*p* < 0.0001) between the TNBS + TOP10 and TNBS + TOP20 groups. The control groups had similar results: TOP20 (11.00 ± 0.71 ng/mg), vehicle (10.00 ± 1.47 ng/mg), ethanol (9.00 ± 0.00 ng/mg), and sham (6.5 ± 0.85 ng/mg).

### 2.3. Measurement of Cytokines

**Figure 5:**
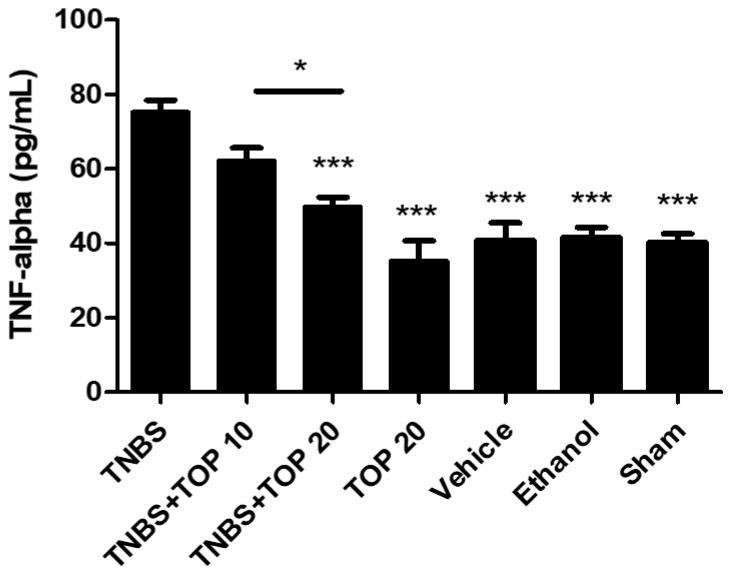
Effect of topiramate treatment on TNF-α concentration. Legend: one-way ANOVA and Tukey’s post-hoc test, * *p* < 0.01 comparing the TNBS + TOP10 group with the TNBS + TOP20 group; *** *p* < 0.0001 compared with TNBS group.

The measurement of the pro-inflammatory cytokine TNF-α allows for the analysis of the inflammatory response and the comparison of the results between all groups (Figure 5). As expected, the TNBS group presented the highest levels of TNF-α (75.28 ± 3.17 pg/mL). Topiramate treatment showed a beneficial effect on this parameter, but only in the highest dose since the TNBS + TOP20 group showed a significant decrease in TNF-α levels in comparison to the TNBS group (49.81 ± 2.58 pg/mL vs. 75.28 ± 3.17 pg/mL, *p* < 0.0001). Nevertheless, there were no significant differences between the TNBS + TOP10 and TNBS groups (62.21 ± 3.45 pg/mL vs. 75.28 ± 3.17 pg/mL). These results indicate a dose-dependent effect of topiramate in the treatment of TNBS-induced colitis, since there were statistically significant differences between the groups treated with different doses of topiramate (*p* < 0.01). Furthermore, the TOP20 (35.25 ± 5.39 pg/mL), sham (40.18 ± 2.33 pg/mL), vehicle (40.78 ± 4.66 pg/mL), and ethanol (41.50 ± 2.80 pg/mL) control groups reduced TNF-α levels significantly more than the TNBS group (75.28 ± 3.17 pg/mL, *p* < 0.0001).

**Figure 6:**
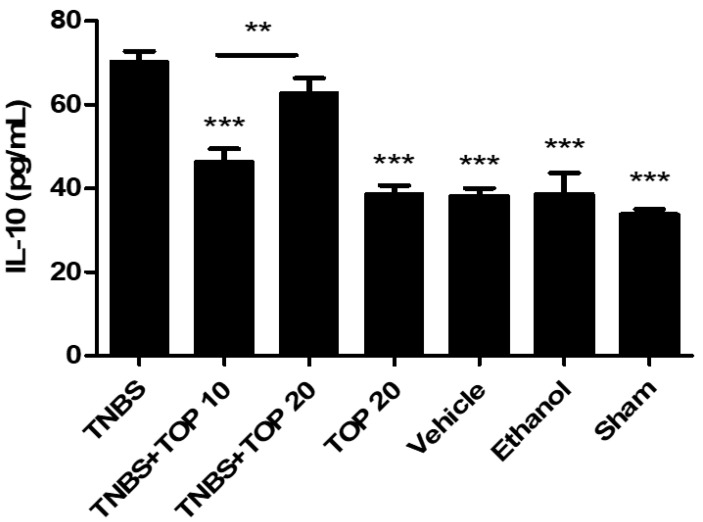
Effect of topiramate treatment on IL-10 concentration. Legend: one-way ANOVA and Tukey’s post-hoc test, ** *p* < 0.001 comparing TNBS + TOP10 group with the TNBS + TOP20 group; *** *p* < 0.0001 compared with the TNBS group.

IL-10 is an anti-inflammatory cytokine that plays a central role in the mucosal immune system, inhibiting pro-inflammatory responses [16]. The determination of IL-10 concentration is critical for comparing results across all experimental groups and validating the results with TNF-α measurements (Figure 6). The TNBS group presented the highest concentration of IL-10 (70.31 ± 2.43 pg/mL). There was a very significant decrease in the concentration of this cytokine in the TNBS + TOP10 group (46.53 ± 2.99 pg/mL) in comparison to the TNBS group (*p <* 0.0001). The TNBS + TOP20 group also showed a decrease in the levels of this cytokine; however, without statistically significant differences compared to the TNBS group (62.81 ± 3.53 pg/mL vs. 70.31 ± 2.43 pg/mL). It was also observed statistically significant differences in IL-10 levels between the TNBS + TOP10 group and the TNBS + TOP20 group (46.53 ± 2.99 pg/mL vs. 62.81 ± 3.53 pg/mL, *p <* 0.001). The TOP20 (38.78 ± 1.92 pg/mL), ethanol (38.60 ± 5.10 pg/mL), vehicle (38.08 ± 1.99 pg/mL), and sham (33.80 ± 1.21 pg/mL) groups demonstrated a significant decrease in IL-10 levels when compared to the TNBS group (*p <* 0.0001).

### 2.4. Microscopial Assessment of Colitis Severity

**Figure 7:**
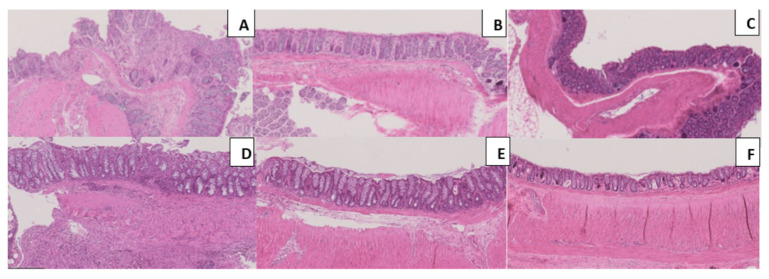
Histopathological analyses of Masson’s trichrome staining 10×. Legend: (**A**) TNBS group; (**B**) ethanol group; (**C**) sham group; (**D**) TNBS + TOP10 group; (**E**) TNBS + TOP20 group; (**F**) TOP20 group.

The differences according to the histopathological evaluation, between the groups, can be represented by the illustrative images present on Figure 7. The histopathological analysis allows the evaluation of colonic injury based on inflammatory cell infiltration and tissue damage. The colons of TNBS group presented a significant infiltration of inflammatory cells, mostly in the mucosa and submucosa layers, foci of ulceration with necrosis, and tissue disruption. In the TOP-treated groups, the level of inflammatory cell infiltration along the area of epithelial ulceration and tissue disruption decreased. TOP showed a significant anti-inflammatory effect when used at the highest dosage, namely at the TNBS + TOP20 group, having the least rate of cell infiltration.

**Figure 8:**
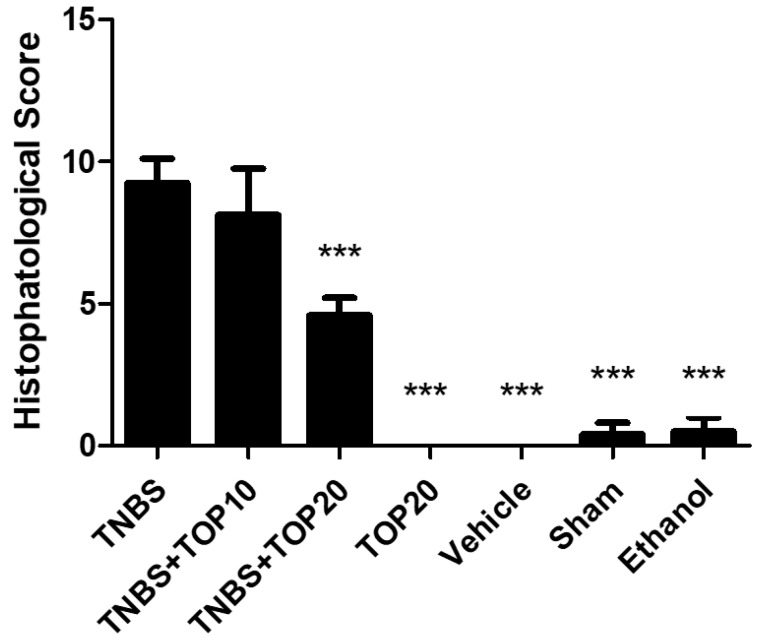
Histopathological score between the groups. Legend: one-way ANOVA and Tuckey’s post-hoc test. *** *p* < 0.0001; compared with TNBS group.

Concerning the microscopical assessment of colitis severity, it was generated a final histopathological score in order to generate a more objective evaluation, which promoted a more objective comparation between the groups (Figure 8). As expected, the animals present in the TNBS group revealed the highest histopathological score, namely 9.25 ± 0.86. Indeed, according to the data obtained in TNBS + TOP20 (4.60 ± 0.61), TOP20 (0.0 ± 0.0), vehicle (0.0 ± 0.0), ethanol (0.50 ± 0.50), and sham (0.40 ± 0.40) groups, it was identified a significant reduction in comparison to the TNBS group (*p* < 0.0001). The TNBS + TOP10 (8.13 ± 1.62) showed a reduction of the histopathological score compared to the TNBS group; however, it was not statistically significant.

## 3. Discussion

The long-term use of corticosteroids or systemic anti-inflammatory drugs, which are commonly used to treat IBD, is associated with severe long-term side effects, and more targeted therapies, such as TNF-α drugs, are very expensive. Topiramate, a potent antiepileptic drug, widely used to treat seizures and migraines, has previously been suggested as an interesting pharmacological approach for the treatment of IBD [8,9,17]. According to some current preclinical studies, there is already some evidence of its beneficial effects on inflammatory diseases, including IBD. This antiepileptic drug, surprisingly, has been described as having superior therapeutic efficacy compared to some approved treatments, among them, the corticosteroid prednisolone. Taking into account the existing literature, topiramate has been demonstrated to significantly reduce symptoms such as diarrhea, swelling, ulceration, and other gross pathological characteristics in animal models of IBD. In this sense, topiramate seems to be a promising pharmacological approach for the treatment of IBD in the future. In this experimental study, we tested its influence on a model of TNBS-induced colitis, by the daily administration of this drug for 14 days.

Firstly, in terms of the manifestations of TNBS-induced colitis by the evaluation of clinical signs, the TNBS group has shown the worst prognosis, with changes in intestinal motility, characterized by the presence of diarrhea and soft stool, and moderate edema on the anus. These results are coincident with the correct induction of experimental chronic TNBS-induced colitis, as described by some authors [18,19,20]. On the other hand, these changes were not observed in the control groups (TOP20, vehicle, ethanol, and sham), which was expected, since the animals were not induced with the disease. In the TNBS + TOP10 and TNBS + TOP20 groups, topiramate normalized the clinical signs, such as stool consistency and anus appearance. However, this drug did not show a significant effect on body weight. Mice with colitis had a progressive increase in body weight throughout the experiment, which is consistent with the results obtained in previous studies [20,21,22,23]. In the third week of the experiment, groups with TNBS-induced colitis showed a decrease in this parameter, which may be related to the fragility of the mice after three TNBS administrations. Nevertheless, a recovery in body weight was observed over the following weeks after starting treatment with topiramate, which was not expected since topiramate has been reported to reduce body weight in humans and is currently used to treat eating disorders and obesity [18,21,24]. However, we believe that this effect of topiramate only occurs after prolonged administrations with higher doses.

In this study, we measured ALP levels to look for evidence of colon inflammation. [25]. Regarding ALP concentration, as expected, the TNBS control group presented the highest concentration, which suggests that the increased levels of ALP observed in this group results from the induction of intestinal inflammation with TNBS [18,25,26]. Furthermore, in comparison to the TNBS group, the control groups TOP20, ethanol, vehicle, and sham had significantly lower levels of this marker. Topiramate showed a beneficial effect on this parameter, it significantly reduced the ALP levels in both the TNBS + TOP10 (*p <* 0.0001) and TNBS + TOP20 groups (*p <* 0.001). These results demonstrate a potential anti-inflammatory effect of topiramate.

In order to evaluate the intensity of the hemorrhagic focus, which can be useful in the diagnosis of colorectal disorders and other potential lesions that are accompanied by bleeding, the fecal hemoglobin was measured [27]. Topiramate showed a positive influence on decreasing the levels of fecal hemoglobin in both doses, in comparison to the TNBS group. Nonetheless, only the highest dose used demonstrated a significant decrease in this parameter (*p <* 0.001). Since there were no significant differences between both groups treated with topiramate, no dose-dependent effect was observed. Although topiramate proved to be beneficial in reducing the concentration of this marker, the values obtained in the groups treated with topiramate were not similar to those obtained in the control groups (TOP20, vehicle, ethanol, and sham), which can be explained by the absence of the disease in these groups.

The measurement of fecal calprotectin permits the evaluation of the accumulation of leukocyte in stool, which is considered as one of the most sensitive non-invasive markers in the distinction of IBD from other functional disorders [15,28]. According to the results obtained, it is possible to conclude that the treatment with TOP was able to significantly reduce the concentration of this marker, in comparison to the TNBS group (*p* < 0.0001). On the other hand, there was identified a dose-dependent effect by TOP, showing an increased beneficial role from this molecule when administrated in higher dosages. In the literature, it is possible to observe that this marker is not considered in several preclinical studies, concerning an IBD context; however, as demonstrated in this experiment, it can be a valuable parameter to be evaluated since it is relatively easy to determine and reveals a high sensitivity.

The pro-inflammatory cytokine TNF-α and the anti-inflammatory cytokine IL-10 were measured to investigate the effect of topiramate on the inflammatory response. Once again, the TNBS group had the highest TNF-α levels, while the control groups TOP20, sham, vehicle, and ethanol had a very significant reduction in TNF-α levels when compared to the TNBS group (*p <* 0.0001). Topiramate has proved to be beneficial in reducing the concentration of TNF-α, but only at the highest dose since the TNBS + TOP20 group showed a significant decrease in the levels of this parameter compared to the TNBS group (*p <* 0.0001), but no significant differences were observed between the TNBS + TOP 10 and the TNBS groups. This finding may suggest a dose-dependent effect of topiramate in the treatment of IBD since there were statistically significant differences between both groups treated with topiramate at different doses (*p <* 0.01). Regarding IL-10, the TNBS group presented the highest value among all groups, which may be linked to the adaptation of the immune system to chronic inflammation of colitis, which results in a greater production of this anti-inflammatory cytokine to combat the disease. Both groups treated with topiramate showed a decrease in levels of this cytokine compared to the TNBS group. However, this decrease was only significant at the lowest dose of topiramate (*p <* 0.001). These observations can be explained by the fact that topiramate reduced the inflammatory response over the 14 days of administration and, consequently, at the end of the experiment, the concentration of this anti-inflammatory cytokine was significantly lower compared to the TNBS group. Taking these findings into account, as expected, it was possible to observe a positive influence of topiramate on the inflammatory response.

Throughout the microscopical evaluation, it was observed a potential beneficial effect by TOP, especially at 20 mg/Kg, translated in the reduction of the histopathological score on the TOP-treated groups in comparison to the TNBS group. However only the highest dose clearly confirms it since there were significant differences (*p <* 0.001). Indeed, there were no data available on the literature that TOP had demonstrated to be capable of significantly attenuating the inflammatory response, concerning the histopathological evaluation.

## 4. Materials and Methods

### 4.1. Drugs and Chemicals

In this study, 2,4,6-trinitrobenzenosulfônico acid (TNBS 1%), topiramate (Topiramato 25 mg tolife^®^), and sodium hydroxide (NaOH) from Sigma Chemical Co. (Lisbon, Portugal) were used. Ketamine (Ketamidor^®^ 100 mg/mL) was purchased from Merial (Lisbon, Portugal). Xylazine 20mg/mL (Sedaxylan^®^) was purchased from Bayer (Lisbon, Portugal). ADVIA^®^ kit was purchased from Siemens Healthcare Diagnostics (Munich, Germany).

### 4.2. Animals

Female CD-1 mice of 6–10 weeks of age, weighing 20–30 g, were obtained from the Institute of Hygiene and Tropical Medicine. The animals were housed in the Faculty of Pharmacy Animal Facility, at the University of Lisbon (FFUL), at 18–23 °C of temperature and 40–60% of humidity, with a controlled 12 h light/dark cycle. Standard polypropylene cages were used to keep the rodents with ad libitum access to food and water. Animal care was in compliance with the Directive 2010/63/EU. The experiment was approved by the Ethics Committee for Animal Experimentation of the Faculty of Pharmacy of the University of Lisbon, 3/2020.

### 4.3. Induction of TNBS-Induced Colitis and Administration Protocol of Topiramate

Mice were kept fasting for 24 h before the induction of experimental colitis. On day 0 of the experiment (induction day), the mice were anesthetized by an intraperitoneal (IP) injection of ketamine 100 mg/kg + xylazine 10 mg/kg. Thereafter, 100 µL of 1% TNBS in 50% ethanol was administered through a catheter, carefully inserted until 4 cm into the colon. Then, mice were kept for 1 min in a Trendelenburg position to avoid reflux [18,29]. This procedure was performed on days 0, 7, 14, 21, and 28 of the experiment, over 5 weeks. From day 21 to day 35, 40 µL of topiramate (TOP) at a dose of 10 mg (TOP10) and 20 mg (TOP20) were administered daily via IP. On day 36, the animals were anesthetized, and blood samples were collected by cardiac puncture for the determination of biochemical markers. Finally, mice were sacrificed by cervical dislocation. The abdomen was opened by a midline incision and the colon was removed, freed from surrounding tissues.

### 4.4. Experimental Groups

The experiment includes 65 mice that were categorized into 7 experimental groups. Two treated groups with the disease, TNBS + TOP10 and TNBS + TOP20, and 5 control groups, TNBS, TOP20, vehicle, sham, and ethanol groups. TNBS + TOP10 group (n = 15) and TNBS + TOP20 group (n = 15) received 1% TNBS, for 5 weeks, and were treated daily with IP administration of 10 mg/kg and 20 mg/kg of topiramate (diluted in NaOH 1 M), respectively. The treatment with TOP began on day 21 of the experiment. TOP20 group (n = 10) received daily 20 mg/kg of topiramate starting on day 21 of the experiment; the vehicle group (n = 10) received daily NaOH 1 M (topiramate vehicle) from day 21; the TNBS group (n = 5) received an intrarectal administration of 100 µL of 1% TNBS from day 0 to day 28; ethanol group (n = 5) received weekly intrarectal administration of 50% ethanol (TNBS vehicle); and a sham group (n = 5) received a 100 µL intrarectal saline solution (NaCl 0.9%).

### 4.5. Monitoring of Clinical Signs

Throughout the entire experiment, the animals were observed daily, monitoring body weight, stool consistency, anus appearance, and survival rate.

### 4.6. Biochemical Markers and Cytokines

The serum was separated from blood samples by centrifugation at 3600 rpm for 15 min and analyzed by an automated clinical chemistry analyzer (ADVIA^®^1200). Serum analysis was performed in order to evaluate ALP, TNF-α, and IL-10. Fecal hemoglobin and fecal calprotectin were also evaluated, using a quantitative method by immunoturbidimetry (Kroma Systems).

### 4.7. Microscopical Evaluation

In order to evaluate colitis severity, a microscopical evaluation of colon tissue was performed. A portion of four centimeters of distal colon tissue was fixed in 10% phosphate-buffered formalin, sectioned at 5 µm, and then stained with hematoxylin and eosin. To increase the possibility of detecting fibrosis, Masson’s trichrome staining was used. The intestinal samples were evaluated by an independent histopathologist, considering several parameters such as tissue loss, epithelial lesions, inflammation grade, fibrosis, and the total extent of the disease [30,31]. In addition to the qualitative microscopic evaluation, a quantitative analysis was also carried out between groups, attributing punctuation between 0 and 4 in each factor, previously referred to, which its sum resulted in a final histopathological score (maximum of 20).

### 4.8. Statistical Analysis

All results were analyzed using GraphPad Prism 5.0 software (GraphPad, San Diego, CA, USA). Statistical significance was determined by one-way ANOVA followed by Tukey’s post-hoc test for multiple comparisons. The results were expressed as the mean ± SEM of N observations, where N represents the number of animals analyzed. A value of *p* < 0.05 was considered significant.

## 5. Conclusions

According to the findings of this study, topiramate showed a beneficial anti-inflammatory effect in the treatment of IBD, as it was able to normalize clinical signs such as stool consistency and anus appearance and attenuate inflammation associated with chronic experimental colitis. Topiramate also had a positive effect on the biomarker’s fecal hemoglobin, fecal calprotectin, and ALP, as well as significantly lowering the concentrations of pro-inflammatory cytokine TNF-α and anti-inflammatory cytokine IL-10.

It was possible to observe a dose-dependent effect of topiramate on the expression of TNF-α. Considering the findings of this experimental study, topiramate has been shown to be a promising pharmacological approach for the treatment of IBD in the future.

## Data Availability

Not applicable.
